# Cortical involvement of lateral trunk flexion and verticality misperception in Parkinson’s disease

**DOI:** 10.1093/braincomms/fcaf040

**Published:** 2025-01-27

**Authors:** Masayuki Kohsaka, Tomoko Oeda, Shigetoshi Takaya, Satoshi Tomita, Kwiyoung Park, Kenji Yamamoto, Hidenao Fukuyama, Hideyuki Sawada

**Affiliations:** Clinical Research Center and Department of Neurology, National Hospital Organization Utano National Hospital, Kyoto 616-8255, Japan; Clinical Research Center and Department of Neurology, National Hospital Organization Utano National Hospital, Kyoto 616-8255, Japan; Department of Neurology and Rehabilitation Medicine, Senri Rehabilitation Hospital, Osaka 562-0032, Japan; Clinical Research Center and Department of Neurology, National Hospital Organization Utano National Hospital, Kyoto 616-8255, Japan; Clinical Research Center and Department of Neurology, National Hospital Organization Utano National Hospital, Kyoto 616-8255, Japan; Clinical Research Center and Department of Neurology, National Hospital Organization Utano National Hospital, Kyoto 616-8255, Japan; Department of Neurology, Yasu City Hospital, Shiga 520-2331, Japan; Clinical Research Center and Department of Neurology, National Hospital Organization Utano National Hospital, Kyoto 616-8255, Japan

**Keywords:** Parkinson’s disease, verticality perception, postural abnormality, temporoparietal association cortices, SPECT

## Abstract

Lateral trunk flexion is a common form of postural abnormality in Parkinson’s disease and could be associated with verticality misperception. However, the mechanisms underlying lateral trunk flexion and verticality misperception in Parkinson’s disease remain unclear. In the current study, we examined whether lateral trunk flexion is associated with verticality misperception in patients with Parkinson’s disease. We also identified the brain regions involved in lateral trunk flexion and altered verticality perception. In this cross-sectional study, we evaluated the verticality perception using the subjective visual vertical test in 81 patients with Parkinson’s disease and 14 age-matched healthy controls. According to the 97.5th percentile upper reference limit of the body tilt angle in the healthy controls, patients with Parkinson’s disease were grouped into 37 patients with lateral trunk flexion and 44 patients without lateral trunk flexion. The mean of absolute subjective visual vertical angles was compared between patients with Parkinson’s disease with lateral trunk flexion, those without lateral trunk flexion, and the healthy controls, and the impact of verticality misperception on lateral trunk flexion was evaluated using multivariate logistic regression models. We further performed a voxel-wise comparison of regional cerebral blood flow using N-isopropyl-p-[^123^I] iodoamphetamine single-photon emission computed tomography (height threshold of *P* < 0.001, uncorrected for multiple comparisons, extent threshold of 100 voxels) to identify the brain regions associated with lateral trunk flexion, and to investigate the relationship between verticality misperception and regional hypoperfusion. The mean of absolute subjective visual vertical angles was larger in patients with Parkinson’s disease with and without lateral trunk flexion than in healthy controls (*P* < 0.001 and *P* < 0.001). Additionally, the subjective visual vertical angle was associated with the presence of lateral trunk flexion (odds ratio 2.25, 95% confidence interval 1.51–3.36, *P* < 0.001). The regional cerebral blood flow in patients with Parkinson’s disease with lateral trunk flexion was decreased in the right inferior parietal lobule, right superior parietal lobule, right superior temporal gyrus, and right dorsal posterior cingulate cortex compared with those without lateral trunk flexion. The subjective visual vertical angle was inversely correlated with regional cerebral blood flow in these regions, except for the dorsal posterior cingulate cortex. Our study reveals that hypofunction in the right temporoparietal association cortices is involved in verticality misperception and the development of lateral trunk flexion in patients with Parkinson’s disease. These results provide insights into potential therapeutic targets for addressing lateral trunk flexion.

## Teaser

Kohsaka et al. report that lateral trunk flexion, a common postural abnormality in Parkinson’s disease, is associated with the changes in verticality misperception and hypofunction in the right temporoparietal association cortices. These results elucidate the neural basis and potential therapeutic targets for lateral trunk flexion in Parkinson’s disease.

## Graphical Abstract

**graphical abstract:**
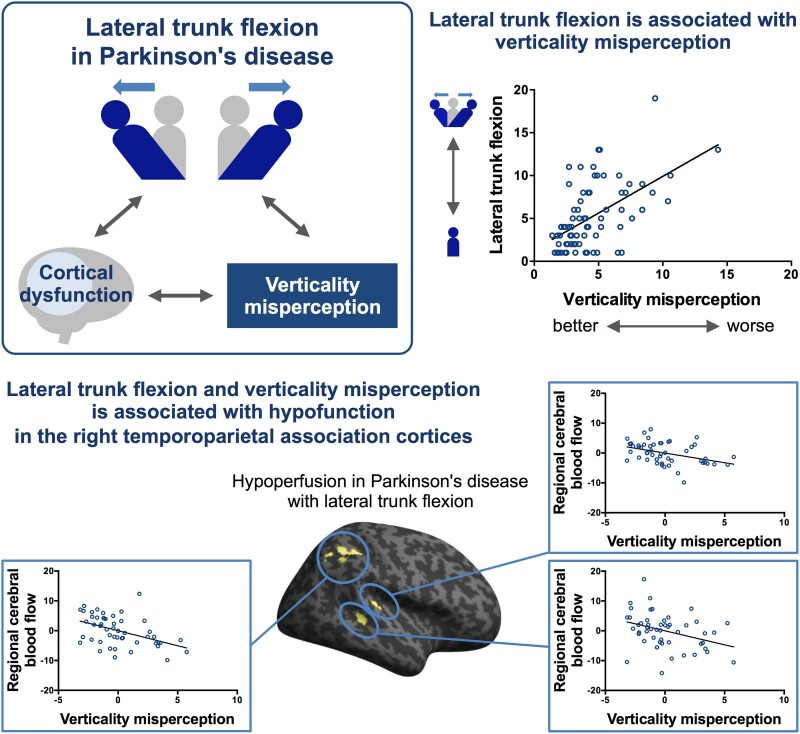

## Introduction

Lateral trunk flexion (LTF) is a common postural abnormality in patients with Parkinson’s disease.^1^ It is an important clinical issue increasing the risk of falls and impairing the patient’s quality of life.^2,3^ However, the pathophysiological mechanisms remain unclear, and efficient treatment approaches have not been established.^1,4^

The appropriate modulation of verticality perception is essential for maintaining a proper upright posture.^5^ Constructing and updating verticality perception depends on accurate processing and integration of multisensory information from the visual, vestibular and somatosensory systems.^6^ Since previous studies have reported that verticality perception is impaired in patients with Parkinson’s disease with LTF,^7,8^ we hypothesized that verticality misperception would interfere with the ability to maintain proper posture in these patients.

Verticality perception is constructed in higher-order cortical processing areas where multisensory information is integrated.^9^ Previous functional MRI and noninvasive brain stimulation studies have shown that the inferior parietal lobule and superior temporal gyrus are crucial in verticality perception in healthy controls.^10-13^ Lateropulsion is a pathological phenomenon often observed in patients with stroke.^14,15^ It is characterized by a lateral tilt of the body toward the side contralateral to the lesion. Neuroimaging studies have demonstrated that lateropulsion is associated with the contralateral lesions in the temporoparietal association cortices.^6,16^ These studies suggest that the temporoparietal association areas are essential for correct verticality perception and appropriate upright posture. However, the brain regions responsible for abnormal posture and verticality misperception in Parkinson’s disease remain to be elucidated. We hypothesized the involvement of cortical regions in abnormal posture and verticality misperception in patients with Parkinson’s disease as well as patients with stroke.

This study defined LTF by comparing the degree of body tilt angle in patients with Parkinson’s disease with that of age-matched healthy controls, aiming for the early detection of mild postural abnormality and facilitating future therapeutic interventions. We assessed verticality perception using the subjective visual vertical (SVV) test and analysed regional cerebral blood flow (rCBF) using perfusion single-photon emission computed tomography (SPECT) in patients with Parkinson’s disease. We examined the association between verticality misperception and LTF and further identified the brain regions involved in these perceptual and postural abnormalities.

## Materials and methods

### Study design

**Figure 1:**
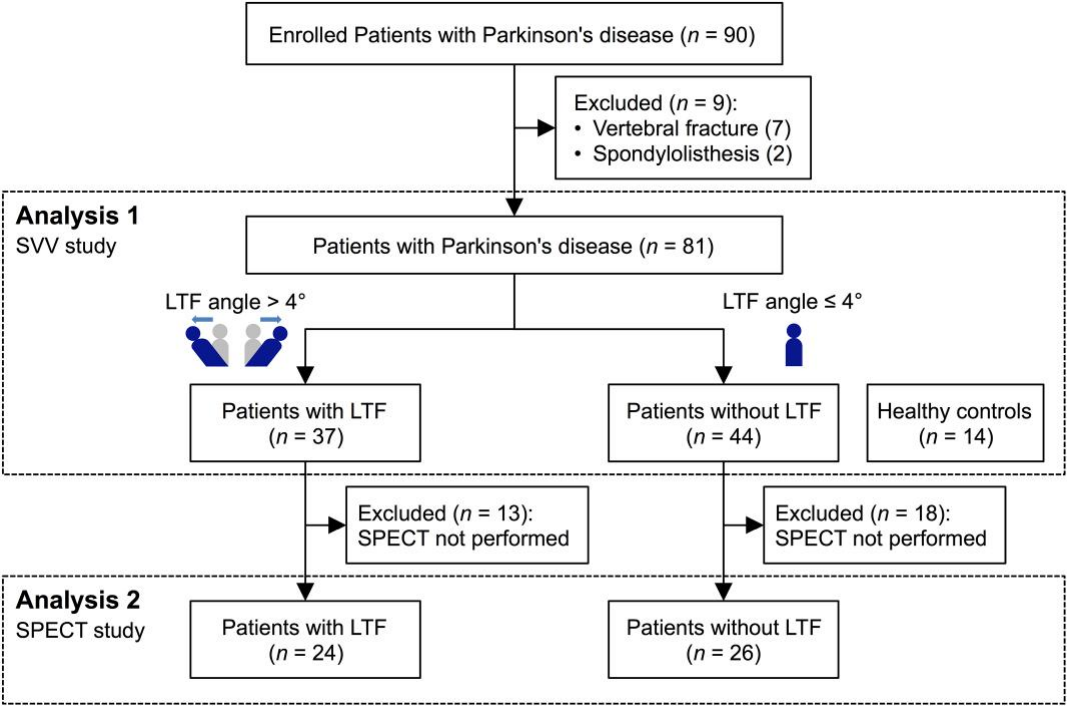
**Flow diagram of the study design.** LTF, lateral trunk flexion; SPECT, single-photon emission computed tomography; SVV, subjective visual vertical.

This cross-sectional study examined whether LTF was associated with the changes in verticality misperception in patients with Parkinson’s disease (Analysis 1) and identified the brain regions involved in postural abnormality and altered verticality perception (Analysis 2; Fig. 1).

### Participants

Ninety consecutive patients with idiopathic Parkinson’s disease were recruited at the National Hospital Organization Utano National Hospital, Kyoto, Japan. All patients met Steps 1 and 2 of the UK Parkinson’s Disease Society BRAIN Bank Diagnostic Criteria, with a modified Hoehn and Yahr (mH-Y) stage from 1 to 4 in the ON state and a Mini-Mental State Examination (MMSE) score of 20 or greater. The diagnosis of idiopathic Parkinson’s disease was made by neurologists with movement disorder expertise based upon clinical history and examination. All patients had a brain MRI. In addition, abnormal postures caused by concomitant conditions were excluded: (1) vertebral fractures or severe spondylolisthesis revealed by spine X-ray or a history of spinal surgery; (2) concomitant neurological diseases that could influence posture, such as stroke^15^; (3) history or clinical signs of vestibular dysfunction, including dizziness, vertigo, spontaneous nystagmus, or diplopia^1,4^; (4) severely impaired visual acuity or visual field defect; (5) signs of somatosensory disturbance in neurological examinations^4^; (6) use of cholinesterase inhibitors, which can induce abnormal posture,^1,4^ within the 6 months before enrolment; and (7) history of stereotaxic neurosurgical procedures, such as pallidotomy and subthalamotomy.^1,4^ We recruited 14 age-matched healthy controls without current or previous neurological or psychiatric diseases.

The institutional bioethics committee approved this study (National Hospital Organization Utano National Hospital approval number: UTA27–34), conducted under the Declaration of Helsinki and the Ethical Guidelines for Medical and Health Research Involving Human Subjects in Japan. Written informed consent was obtained from all participants.

### Clinical assessment

Demographics and clinical information were collected from medical records, including age, sex, Parkinson’s disease duration and medications. Levodopa equivalent daily dose (LEDD) was calculated as previously described.^17^ Motor function was evaluated using the Unified Parkinson’s Disease Rating Scale part III (UPDRS-III), and disease severity was classified according to the mH-Y stage. In patients with motor fluctuations, the UPDRS-III score and mH-Y stage were evaluated in the ON state. Experienced neuropsychologists assessed global cognitive function using the MMSE.

### LTF angle measurement

**Figure 2:**
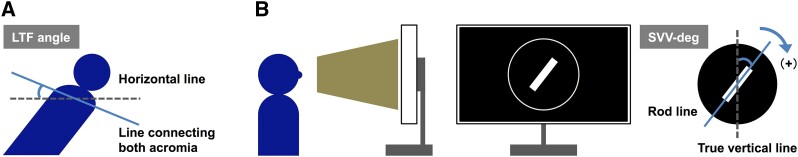
**LTF angle and verticality perception assessments.** (**A**) LTF angle assessment. The LTF angle was defined as the angle between the line connecting both acromia and the horizontal line. This angle was assessed using a dorsal view photograph of each participant. (**B**) Verticality perception assessment. A participant was seated upright in a dimly lit room and instructed to look at a display. The rod was presented on the monitor at a randomly selected angle between −30° and +30°, rotating alternately clockwise and counterclockwise during the assessment. The angle between the true vertical and the rod line perceived by the participant as vertical was recorded 10 times. The mean of the 10 test results was defined as SVV-deg in each participant. LTF, lateral trunk flexion; SVV, subjective visual vertical.

A photograph from a dorsal view of each participant in the upright position was taken to measure the LTF angle, defined as the angle formed by the line connecting both acromia and the horizon (Fig. 2A). In patients with motor fluctuations, the LTF angle was measured in the ON state. We defined an LTF angle larger than the 97.5th percentile upper reference limit in the age-matched healthy controls as abnormal in this study.

### Verticality perception assessment

We assessed verticality perception using the SVV test.^7,8,18^ Each participant was seated upright with their head in a vertical position on a chinrest to minimize posture bias. In a dark room, participants looked at the LED display, located 30 cm in front of their eyes, with a cylinder-shaped shield (29 cm in diameter) that conceals information regarding the verticality and horizontality of the surroundings (Fig. 2B). The LED screen was positioned horizontally, with the centre of the monitor at the same level as the participant’s eyes. In each trial, a white rod (10 cm long, 1 cm wide, and 18.9° of visual angle) on a black background was displayed on the monitor. The rod was presented at a specific, randomly selected angle between −30° (counterclockwise) and 30° (clockwise) and rotated clockwise and counterclockwise alternately at a velocity of 1°/s in each trial. Participants were requested to report when they felt the rod was vertical, and the angle was collected as a signed value (clockwise, positive and counterclockwise, negative; Fig. 2B). The SVV test was repeated 10 times at 1-min intervals to minimize intrasubject variation.^18^

The mean of the 10 test results for each participant was defined as SVV-deg. Additionally, the mean of the absolute values of the angles from each test (|SVV-deg|) was used to evaluate the magnitude of verticality misperception.^18,19^ In patients with motor fluctuations, all assessments were performed in the ON state.

### Sample size calculation

Based on a previous study,^7^ we estimated that 85 patients with Parkinson’s disease would be needed to provide 80% power to detect the difference in |SVV-deg| of at least 3° between patients with Parkinson’s disease with and without LTF, with a two-sided alpha level of 0.05.

### Statistical analysis of the association between verticality misperception and LTF (Analysis 1)

We compared the clinical characteristics of patients with Parkinson’s disease with and without LTF. Scale variables were statistically tested using the Mann–Whitney U-test and Student’s *t*-test for data with non-Gaussian and Gaussian distribution, respectively. The χ^2^ test was used to compare categorical variables.

The relationship between the direction of SVV-deg (positive or negative)^18,19^ and that of LTF was evaluated using the χ^2^ test in patients with LTF to investigate whether the direction of verticality misperception was consistent with that of body tilt. The correlation between |SVV-deg| and LTF angle was analysed using Spearman’s rank correlation test. We also evaluated the correlation between total rigidity scores in UPDRS-III and LTF angle using Spearman’s rank correlation test because previous reports suggested that rigidity might be associated with the mechanisms of LTF.^1,4,20^

We compared |SVV-deg| between the three groups (patients with LTF, patients without LTF and healthy controls) using the Kruskal–Wallis test with the Dunn–Bonferroni post hoc test for multiple comparisons.

The impact of |SVV-deg| on the presence of LTF was evaluated using a multivariate logistic regression analysis, with the odds ratio (OR) calculated as the association strength. Because the mH-Y stage and ongoing combination therapy with levodopa and dopamine agonists are reported to be associated with LTF,^2^ these variables were incorporated in the multivariate models. Additionally, other possible predictive variables were tested. A previous report indicated that patients with Parkinson’s disease with LTF had a longer disease duration and were older than patients without LTF^2^; therefore, we assessed the association between five predictive variables (age, Parkinson’s disease duration, mH-Y stage, pharmacological therapy and |SVV-deg|) and LTF using univariate models. First, we assessed multicollinearity between the possible predictive variables using Spearman’s correlation coefficient. Factors associated with the presence of LTF in the univariate models were picked up as possible predictive variables in the multivariate models. Next, we incorporated the factors into a multivariate model and estimated the adjusted OR using a 95% confidence interval (CI) for the |SVV-deg| for the presence of LTF in a multivariate model. All statistical analyses were performed using IBM SPSS Statistics version 21.0 (IBM, Armonk, NY, USA) and GraphPad Prism version 6.0b (GraphPad Software, San Diego, CA, USA). Statistical significance was defined as a *P* value < 0.05.

### SPECT data acquisition

We examined the patients who consented to N-isopropyl-p-[^123^I] iodoamphetamine (IMP)-SPECT imaging. To evaluate rCBF, we collected SPECT images using a 2-head rotating gamma camera (Symbia E Dual Head System, Siemens, Erlangen, Germany) with a fan-beam collimator. Patients were scanned while lying supine with their eyes closed in a dimly lit environment with minimal background noise. One hundred eleven MBq of IMP (Nihon Mediphysics, Takarazuka, Japan) was administered intravenously to the patients. Data acquisition began 15 min after the injection and continued for 18.1 min. The 128 × 128 matrix images were prefiltered using a Butterworth filter with order 8 and a cut-off frequency of 0.32 cycle/pixel and reconstructed using a Ramp back-projection filter. Attenuation correction was applied using Chang’s method (attenuation coefficient of 0.07/cm).

### SPECT data analysis (Analysis 2)

IMP-SPECT images were obtained within 3 months around the SVV tests, and the data were analysed voxel-wise using SPM8 (https://www.fil.ion.ucl.ac.uk/spm/). The images were spatially normalized to fit customized SPECT templates coregistered onto the Montreal Neurological Institute space. We visually confirmed the absence of spatial normalization errors. A Gaussian filter with a full width at a half maximum of 16 mm was used to smooth the spatially normalized images. Each voxel count was normalized to the total count of the whole brain using proportional scaling. To investigate regional hypofunction associated with LTF in Parkinson’s disease, we used a two-sample *t*-test for voxel-wise group comparison of SPECT images between patients with Parkinson’s disease with and without LTF. We obtained *T*-map of voxels with a height threshold of *P* < 0.001 (uncorrected for multiple comparisons), and clusters larger than 100 were considered statistically significant areas.^21^ The Montreal Neurological Institute coordinates of the local maxima from the *t*-statistics were transformed to Talairach coordinates using appropriate converter software (https://bioimagesuiteweb.github.io/webapp/mni2tal.html), and the corresponding brain areas were identified.

### Correlations between verticality misperception and rCBF

To further examine the relationship between verticality perception and the degree of hypofunction in brain areas associated with LTF, we performed correlation analyses between |SVV-deg| and the rCBF of the brain regions where regional uptake was statistically lower in patients with LTF than in those without LTF. Because age and disease progression degrade verticality perception,^22,23^ we performed partial correlation analyses for patients with Parkinson’s disease with and without LTF using age and mH-Y stage as control variables. The Bonferroni correction was used for multiple comparisons.

## Results

### Clinical characteristics

Of the 90 patients with Parkinson’s disease, nine patients were excluded because of previous vertebral compression fractures (*n* = 7) or spondylolisthesis (*n* = 2). The remaining 81 patients and 14 age-matched healthy controls were included in Analysis 1 (Fig. 1). Because the 97.5th percentile upper reference limit of the body tilt angle in the healthy controls was 4°, 37 patients with an LTF angle > 4° were designated as Parkinson’s disease with LTF, and 44 patients with an LTF angle ≤ 4° as Parkinson’s disease without LTF (Supplementary Fig. 1). The definition of LTF in this study was consistent with the cut-off value for LTF angle in a previous study.^24^ The characteristics of the participants in these groups are shown in Table 1. Age, sex, Parkinson’s disease duration, LEDD (mg/day), LEDD (mg/day) of dopamine agonists, prevalence of levodopa monotherapy, combination therapy with levodopa and dopamine agonists and MMSE scores were similar in patients with and without LTF. The UPDRS-III score and mH-Y stage were worse in patients with LTF than those without LTF but with no significant difference (*P* = 0.16 and 0.16, respectively).

**Table 1:** Characteristics of participants for Analysis 1

	Patients with LTF (*n* = 37)	Patients without LTF (*n* = 44)	Healthy controls (*n* = 14)	*P* value^a^	*P* value^b^
LTF angle, median (IQR), degrees	8 (6–10)	2 (1–3)	2 (1–3)	<0.001	<0.001
Age, mean (SD), years	72.2 (7.0)	71.0 (6.9)	71.9 (5.5)	0.45	0.72
Sex, *n* (%)					
Male	14 (37.8)	13 (31.0)	7 (50.0)	0.43	0.71
Female	23 (62.2)	31 (69.0)	7 (50.0)		
Duration of Parkinson’s disease, mean (SD), years	9.2 (3.8)	8.5 (4.7)		0.46	
UPDRS-III score, mean (SD)	22.5 (8.2)	20.0 (8.0)		0.16	
mH-Y stage, *n* (%)					
<3	18 (48.6)	28 (63.6)		0.16	
≥3	19 (51.4)	16 (36.4)			
Direction of LTF, *n* (%)					
Rightward	21 (56.8)				
Leftward	16 (43.2)				
LEDD, median (IQR), mg/day	583 (475–764)	550 (393–794)		0.61	
Dopamine agonists dose, median (IQR), mg (LEDD)/day	50 (0–135)	39 (0–150)		0.75	
Levodopa monotherapy, *n* (%)	14 (37.8)	19 (43.2)		0.63	
Combination therapy with levodopa and dopamine agonists, *n* (%)	23 (62.2)	25 (56.8)		0.63	
MMSE score, median (IQR)	29.0 (25.0–30.0)	29.0 (27.0–30.0)		0.37	

IQR, interquartile range; LEDD, levodopa equivalent daily dose; LTF, lateral trunk flexion; mH-Y, modified Hoehn and Yahr; MMSE, Mini-Mental State Examination; SD, standard deviation; UPDRS-III, Unified Parkinson’s Disease Rating Scale part III.

^a^Comparison between patients with Parkinson’s disease with and without LTF.

^b^Comparison between patients with Parkinson’s disease and the healthy controls.

### Relationship between the direction of verticality misperception and LTF

**Figure 3:**
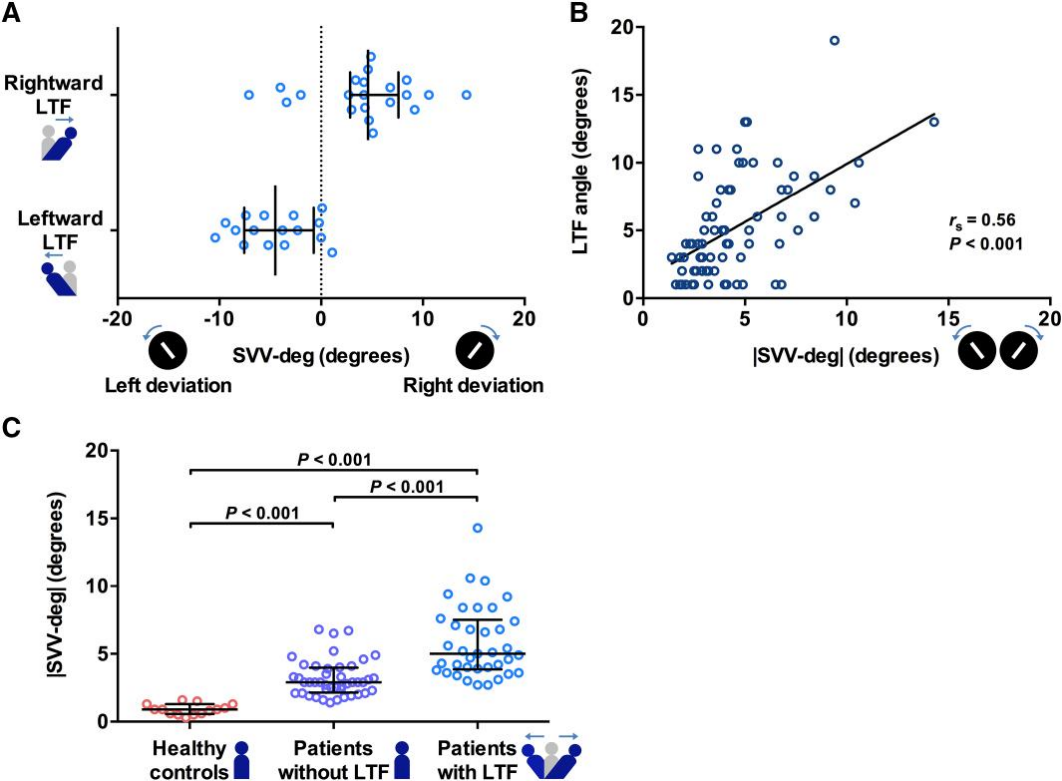
**Direction (SVV-deg) and magnitude (|SVV-deg|) of verticality misperception in patients with Parkinson’s disease.** (**A**) The direction of verticality misperception (SVV-deg deviation) was consistent with the side of LTF in 81.1% of patients with Parkinson’s disease with LTF (*n* = 37). There was a relationship between the SVV-deg deviation and LTF directions (*P* < 0.001). (**B**) The magnitude of verticality misperception (|SVV-deg|) was positively correlated with the LTF angle in patients with Parkinson’s disease (*r*_s_ = 0.56, *P* < 0.001). (**C**) The |SVV-deg| was larger in patients with Parkinson’s disease even without LTF (*n* = 44) than in healthy controls (*n* = 14) and was much larger in patients with LTF (*n* = 37) [*F*(2,92) = 39.72, *P* <0.001]. The statistical analyses were performed using the χ^2^ test (**A**) and the Kruskal–Wallis test with the Dunn–Bonferroni post hoc test for multiple comparisons (**C**). The bars represent the median with the interquartile range. LTF, lateral trunk flexion; SVV-deg, the mean value of the angles that a participant felt to be vertical in subjective visual vertical tests; |SVV-deg|, the mean of the absolute values of the angles from each test.

The direction of SVV-deg was consistent with the direction of LTF in 81.1% (30 out of 37) of the patients with rightward (*n* = 21) and leftward LTF (*n* = 16; Fig. 3A). A strong association was observed between the SVV-deg and LTF directions (*P* < 0.001).

### Correlation between the LTF angle and magnitude of verticality misperception or degree of rigidity

The |SVV-deg| was significantly correlated with the LTF angle in patients with Parkinson’s disease, including those without LTF (*r*_s_ = 0.56, *P* < 0.001; Fig. 3B). The total rigidity score was not associated with the LTF angle in this cohort (*r*_s_ = 0.08, *P* = 0.47; Supplementary Fig. 2).

### Magnitude of verticality misperception in patients with Parkinson’s disease and healthy controls

The |SVV-deg| [median (interquartile range)] was 5.0° (3.9°–7.5°) in patients with LTF, 2.9° (2.2°–4.0°) in those without LTF, and 0.9° (0.6°–1.3°) in healthy controls. The |SVV-deg| was significantly larger in patients with LTF than in those without LTF (*P* < 0.001) and healthy controls (*P* < 0.001; Fig. 3C). Although no differences were observed in the LTF angle between patients without LTF and healthy controls (*P* = 0.82; Table 1), the |SVV-deg| was significantly larger in patients without LTF than in healthy controls (*P* < 0.001).

### Relationship between the magnitude of verticality misperception and LTF

The univariate regression model demonstrated that the |SVV-deg| was significantly associated with LTF (OR 2.23, 95% CI 1.51–3.28 per degree in |SVV-deg|, *P* < 0.001; Supplementary Table 1). In contrast, age, Parkinson’s disease duration and medications were not associated with LTF, and the mH-Y stage was weakly associated with LTF (*P* = 0.18). As described in the Materials and methods, since the mH-Y stage and combination therapy with levodopa and dopamine agonists are considered predictive factors for LTF,^2^ they were incorporated in the multivariate regression model. The model showed that the |SVV-deg| was significantly associated with LTF (OR 2.25, 95% CI 1.51–3.36 per degree in |SVV-deg|, *P* < 0.001; Table 2). However, in this model, severe mH-Y stage and combination therapy with levodopa and dopamine agonists were not significantly associated with LTF.

**Table 2:** Association of clinical factors with LTF in Parkinson’s disease

Predictive factors	Adjusted OR	95% CI	*P* value
|SVV-deg|, degree	2.25	1.51–3.36	<0.001
mH-Y stage, ≥3 versus <3	0.82	0.26–2.55	0.73
Combination therapy with levodopa and dopamine agonists versus Levodopa monotherapy	1.12	0.37–3.41	0.85

CI, confidence interval; LTF, lateral trunk flexion; mH-Y, modified Hoehn and Yahr; OR, odds ratio; |SVV-deg|, the mean of the absolute values of the angles that a participant felt to be vertical in each subjective visual vertical test.

OR was calculated by a multivariate logistic regression model.

### rCBF changes in patients with Parkinson’s disease with LTF

**Figure 4:**
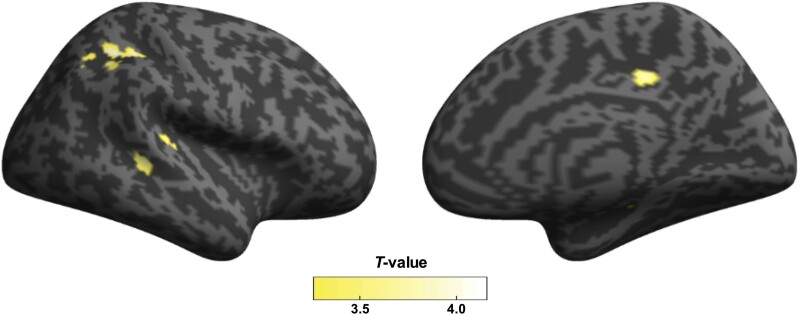
**Distributions of hypoperfusion in patients with Parkinson’s disease with LTF.** Brain regions showing a significant decrease (yellow) in rCBF in patients with Parkinson’s disease with LTF (*n* = 24) compared with patients without LTF (*n* = 26). The areas with a statistically significant reduction in the right hemisphere are shown in the lateral (left) and medial (right) views. Patients with LTF showed reduced rCBF in the right inferior parietal lobule, superior parietal lobule, superior temporal gyrus and dorsal posterior cingulate cortex [*t*(48) = 4.13, *P* < 0.001; *t*(48) = 4.03, *P* < 0.001; *t*(48) = 3.70, *P* < 0.001; and *t*(48) = 3.86, *P* < 0.001, respectively]. No significant areas were found in the left hemisphere. Voxel-wise group comparison of SPECT images was performed using a two-sample *t*-test (height threshold of *P* < 0.001, uncorrected for multiple comparisons, extent threshold of 100 voxels). LTF, lateral trunk flexion; rCBF, regional cerebral blood flow; SPECT, single-photon emission computed tomography.

In Analysis 2, IMP-SPECT images were obtained in 50 patients (24 with LTF and 26 without LTF; Fig. 1). No significant differences were observed between the demographic variables of the two groups (Supplementary Table 2). Compared with patients without LTF, the rCBF in patients with LTF was significantly reduced in the right inferior parietal lobule [IPL, Brodmann area (BA) 39], right superior parietal lobule (SPL, BA 7), right superior temporal gyrus (STG, BA 22) and right dorsal posterior cingulate cortex (dPCC, BA 31; Table 3 and Fig. 4). No voxels survived family-wise error correction at *P* < 0.05 (Supplementary Fig. 3).

**Table 3:** Brain regions showing significantly decreased perfusion in patients with Parkinson’s disease with LTF

Brain region	BA	Side	Coordinate of peak			Cluster size	*T* value
			*x*	*y*	*z*		
Inferior parietal lobule	39	Right	56	−62	46	586	4.13
Superior parietal lobule	7	Right	36	−44	44		4.03
Superior temporal gyrus	22	Right	46	−32	6	171	3.70
Dorsal posterior cingulate cortex	31	Right	6	−28	44	120	3.86

BA, Brodmann area; LTF, lateral trunk flexion.

### Correlations between the magnitude of verticality misperception and rCBF

**Figure 5:**
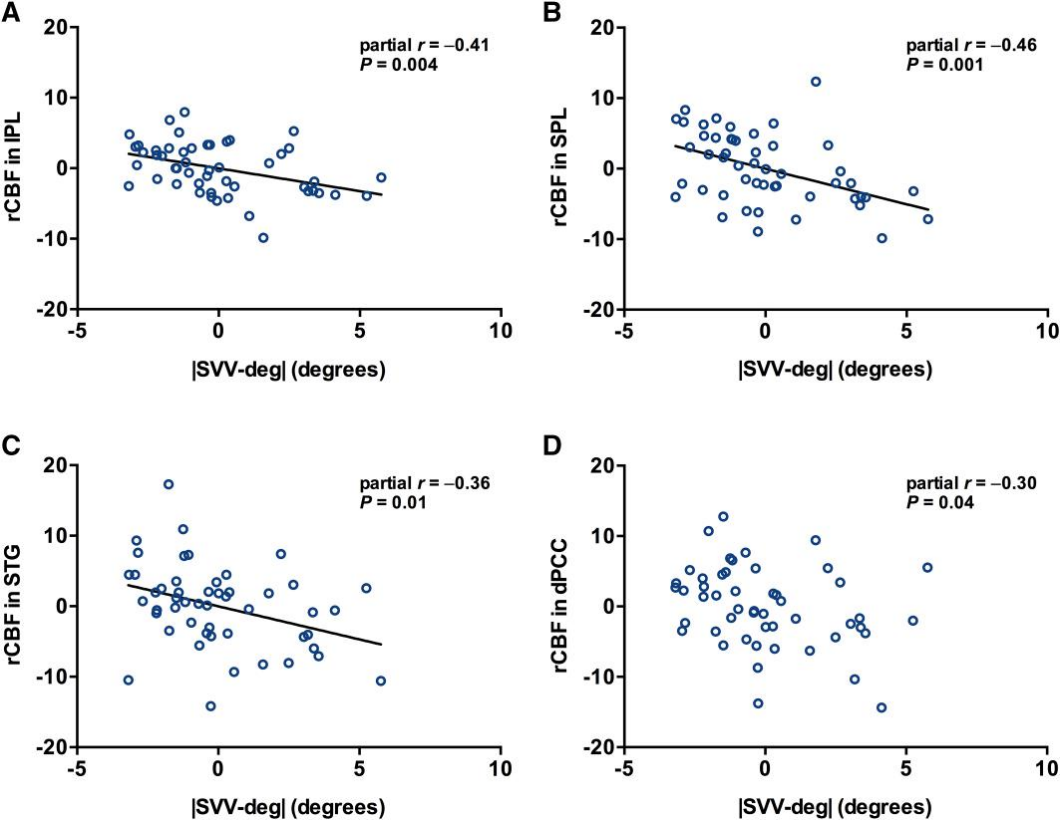
**Correlations between the magnitude of verticality misperception and rCBF.** The magnitude of verticality misperception (|SVV-deg|) was negatively correlated with the rCBF in the brain regions where regional uptake was statistically reduced in patients with Parkinson’s disease with LTF (*n* = 24) and without LTF (*n* = 26). (**A–D**) The partial correlation analyses were performed with age and modified Hoehn and Yahr stage as control variables. The correlations in these brain regions survived the Bonferroni correction for multiple comparisons except for the right dPCC (*P* < 0.05/4). dPCC, dorsal posterior cingulate cortex; IPL, inferior parietal lobule; LTF, lateral trunk flexion; rCBF, regional cerebral blood flow; SPL, superior parietal lobule; STG, superior temporal gyrus; |SVV-deg|, the mean of the absolute values of the angles that a participant felt to be vertical in each subjective visual vertical test.

The correlation between |SVV-deg| and rCBF was investigated using age and mH-Y stage as the control variables. There was a statistically significant correlation between |SVV-deg| and the rCBF in the right IPL, SPL and STG (partial *r* = −0.41, *P* = 0.004; partial *r* = −0.46, *P* = 0.001 and partial *r* = −0.36, *P* = 0.01, respectively), with the Bonferroni correction for multiple comparisons (*P* < 0.05/4; Fig. 5A−C). The |SVV-deg| was also significantly correlated with the rCBF in the right dPCC (partial *r* = −0.30, *P* = 0.04), but no correlation was observed after the Bonferroni correction (*P* > 0.05/4; Fig. 5D).

## Discussion

Verticality perception, which is involved in maintaining a correct posture,^5^ was impaired in patients with Parkinson’s disease with LTF in our study. The multivariate logistic regression analysis demonstrated that the severity of verticality misperception was a significant factor in the presence of LTF in Parkinson’s disease. These results are consistent with previous reports.^7,8,23,25^ Furthermore, our study revealed that impaired verticality perception was present even in patients without LTF whose LTF angles were identical to healthy controls. These data suggest that impaired verticality perception, which may be a possible contributing factor to LTF, could precede the development of LTF in Parkinson’s disease. Despite an impaired verticality perception, normal upright posture was maintained in patients without LTF, suggesting that compensatory mechanisms, such as attention,^26^ might control posture correctly. However, LTF might appear when verticality perception is further impaired, in addition to other contributing factors such as aging and disease progression,^2,22,23^ and compensatory mechanisms no longer work effectively.

The current neuroimaging analyses showed that LTF was associated with hypoperfusion in the right IPL, STG and SPL. Furthermore, the degree of hypoperfusion in these areas was related to the severity of verticality misperception in Parkinson’s disease. Previous reports have shown that verticality perception is associated with the right temporoparietal junction and the adjacent cortical and subcortical regions, including the parietal, temporal and occipital cortices and the superior and inferior longitudinal fasciculus.^6,27^ These brain regions are associated with sensory systems necessary for postural control.^6,28,29^ Among them, the temporoparietal association cortices, including the IPL, STG and SPL, are key cortical areas in integrating multisensory signals from the visual, vestibular and somatosensory systems.^6,30-32^ The integration of multisensory information is essential to construct verticality perception in postural control.^14,26,32,33^ This process involves a delicate balance among the visual, vestibular and somatosensory systems, which dynamically adapt to ensure proper posture.^26,32,33^ However, in individuals with Parkinson’s disease, each sensory modality is impaired, disrupting the equilibrium among sensory inputs and compromising the integration of multisensory modalities.^33,34^ Our results are consistent with the notion that impairment of cross-modal sensory integration in the temporoparietal association cortices is related to verticality misperception in patients with Parkinson’s disease with LTF.

In this study, the four regions associated with LTF in Parkinson’s disease are all located in the right cerebral hemisphere. Verticality misperception was correlated with hypoperfusion in the IPL, STG and SPL. According to an functional MRI study in healthy controls, the IPL and STG in the right hemisphere are activated during the verticality perception task.^10^ Transcranial magnetic stimulation to the right IPL and SPL induces changes in verticality perception in healthy controls.^11-13^ Additionally, verticality perception is altered by inhibitory transcranial direct current stimulation over the right temporoparietal region rather than the left.^35,36^ In patients with stroke, previous structural MRI studies showed that abnormal verticality is associated with cortical lesions in the right temporoparietal association area.^27,37^ In patients with lateropulsion after stroke, impairment of verticality perception is more frequent and severe in the right hemispheric lesions than in the left side.^14,38^ The right hemispheric dominance associated with verticality perception and postural control could be attributed to functional lateralization in the temporoparietal association cortices for visuospatial perception.

The interaction between the basal ganglia and the cerebral cortex, including the temporoparietal association area, is functionally altered in Parkinson’s disease. A resting-state functional MRI study indicates that the functional connectivity between the right IPL and the right putamen is altered even in the early stages of Parkinson’s disease.^39^ These alterations in corticostriatal connectivity may underlie abnormal sensorimotor integration. These results suggest that the right temporoparietal association cortices could be functionally vulnerable regions, related to verticality misperception and postural abnormality in Parkinson’s disease.

Our data indicated that LTF was associated with hypoperfusion in the right dPCC; however, verticality misperception was not correlated with hypoperfusion in this region. The right dPCC has functional connectivity with attentional networks^40^ and a close fibre connection with the IPL.^41^ In this pathway, attention may influence the modulation of multisensory processing involved in postural control.^26,42^ Indeed, LTF is associated with attentional impairment in Parkinson’s disease.^43^ Thus, dysfunction in the right dPCC might not be directly related to verticality misperception but may affect posture through attentional changes in patients with Parkinson’s disease with LTF.

For the mechanisms underlying LTF, abnormal activity in the motor system should be considered, along with changes in the sensory and attentional systems. Increased trunk muscle tone, such as rigidity and dystonia, have been suggested as possible contributing factors to LTF in the central mechanisms.^1,4,20^ However, the degree of rigidity was not associated with LTF in the current cohort. In previous studies, electromyographic findings indicated continuous muscle activity compatible with dystonic contraction in the unilateral paraspinal muscles but with inconsistency.^44-46^ Regarding the peripheral mechanisms, imaging studies using computed tomography and MRI have indicated that the paraspinal muscles atrophy with fatty degeneration is more prevalent on the leaning side.^46,47^ These muscular changes are assumed to be the consequence of disuse and secondary degeneration caused by stretching stress.^46^ Thus, whether trunk rigidity, dystonia and musculoskeletal changes could be the primary cause of LTF remains controversial.

Finally, we discuss the clinical implications of this study. In this study, verticality misperception was observed even before LTF appeared and was associated with the presence of LTF, suggesting that the early intervention to correct verticality misperception may prevent the development of LTF and progression to an irreversible condition.^3,4^ The interventions include rehabilitation programs specifically aimed at correcting altered verticality perception, such as the self-correction of abnormal posture using visual feedback training.^48^ Additionally, our study revealed the cortical regions associated with LTF in patients with Parkinson’s disease. In patients with lateropulsion following stroke, transcranial direct current stimulation over the right temporoparietal region improved postural control.^49^ Noninvasive neuromodulatory techniques, such as transcranial direct current stimulation and transcranial magnetic stimulation, may improve postural abnormality in patients with Parkinson’s disease combined with rehabilitation programs.

This study has some limitations. First, our study was designed to elucidate the association of LTF with verticality misperception, and the number of subjects may be insufficient to demonstrate the association with disease severity and Parkinson’s disease medication, which was found in a previous study.^2^ Second, we did not evaluate in detail peripheral vestibular function, which may also affect verticality perception. However, patients with clinical manifestations of inner ear dysfunction were excluded to minimize the effect. Third, the interval between SVV and SPECT evaluations was a maximum of 3 months. However, given the presumed gradual nature of changes in verticality perception and rCBF associated with LTF, any potential effects of this time gap are expected to be minimal. Fourth, we employed rCBF measured by SPECT as a surrogate marker to assess regional hypofunction in the brain. However, the relatively low sensitivity of SPECT in assessing brain function^50^ might have influenced the observed weak correlations between the magnitude of verticality misperception and rCBF in the partial correlation analyses. Finally, our study was cross-sectional, and we did not determine the causal relationship between abnormal verticality perception and LTF in Parkinson’s disease. Prospective studies with a larger cohort to examine the factors affecting postural abnormality are needed to resolve these limitations.

## Conclusion

We demonstrated that verticality misperception was present before the appearance of LTF and was associated with LTF in Parkinson’s disease. Hypofunction in the right temporoparietal association cortices might contribute to alterations in the construction of verticality perception, resulting in LTF. Our findings could facilitate the early detection of LTF and the development of new treatment strategies.

## Supplementary Material

fcaf040_Supplementary_Data

## Data Availability

The data supporting the findings of this study are available upon reasonable request to the institutional bioethics committee.
