# Supplementary material for: Cortical involvement of lateral trunk flexion and verticality misperception in Parkinson’s disease
Source: Brain Commun. 2025 Jan 27;7(1):fcaf040. doi: 10.1093/braincomms/fcaf040 (PMC11806416; doi:10.1093/braincomms/fcaf040)
Supplement: fcaf040_Supplementary_Data [file fcaf040_supplementary_data.pdf]

Supplementary Tables

Supplementary Table 1. Clinical and demographic variables associated with LTF in univariate logistic regression analysis

Predictive variables	Unadjusted OR	95% CI	*P* value
|SVV-deg|, degree	2.23	1.51–3.28	< 0.001
mH-Y stage, ≥ 3 vs < 3	1.85	0.76–4.50	0.18
Combination therapy with levodopa and dopamine agonists vs Levodopa monotherapy	1.25	0.51–3.05	0.63
Age, 5 years	1.13	0.82–1.56	0.44
Duration of Parkinson's disease, 5 years	1.21	0.72–2.02	0.47

CI, confidence interval; LTF, lateral trunk flexion; mH-Y, modified Hoehn and Yahr; OR, odds ratio; |SVV-deg|, the mean of the absolute values of the angles that a participant felt to be vertical in each subjective visual vertical test.
OR was calculated by a univariate logistic regression model.

Supplementary Table 2. Characteristics of participants for Analysis 2

	Patients with LTF (*n* = 24)	Patients without LTF (*n* = 26)	*P* value
LTF angle, median (IQR), degrees	8 (6–10)	3 (1–4)	< 0.001
Age, mean (SD), years	72.2 (7.0)	70.4 (6.5)	0.45
Sex, *n* (%)			
Male	10 (41.7)	9 (34.6)	0.61
Female	14 (58.3)	17 (65.4)	
Duration of Parkinson's disease, mean (SD), years	9.2 (3.8)	8.6 (5.1)	0.46
UPDRS-III score, mean (SD)	22.5 (8.2)	21.2 (7.4)	0.16
mH-Y stage, *n* (%)			
< 3	11 (45.8)	16 (61.5)	0.27
≥ 3	13 (54.2)	10 (38.5)	
Direction of LTF, *n* (%)			
Rightward	13 (54.2)	-	-
Leftward	11 (45.8)		
LEDD, median (IQR), mg/day	575 (475–769)	550 (402–799)	0.98
Dopamine agonists dose, median (IQR), mg (LEDD)/day	38 (0–150)	20 (0–150)	0.59
Levodopa monotherapy, *n* (%)	10 (41.7)	13 (50.0)	0.56
Combination therapy with levodopa and dopamine agonists, *n* (%)	14 (58.3)	13 (50.0)	0.56
MMSE score, median (IQR)	28.0 (22.0–29.3)	28.5 (27.0–30.0)	0.38

IQR, interquartile range; LEDD, levodopa equivalent daily dose; LTF, lateral trunk flexion; mH-Y, modified Hoehn and Yahr; MMSE, Mini-Mental State Examination; SD, standard deviation; UPDRS-III, Unified Parkinson's Disease Rating Scale part III.

Supplementary Figures

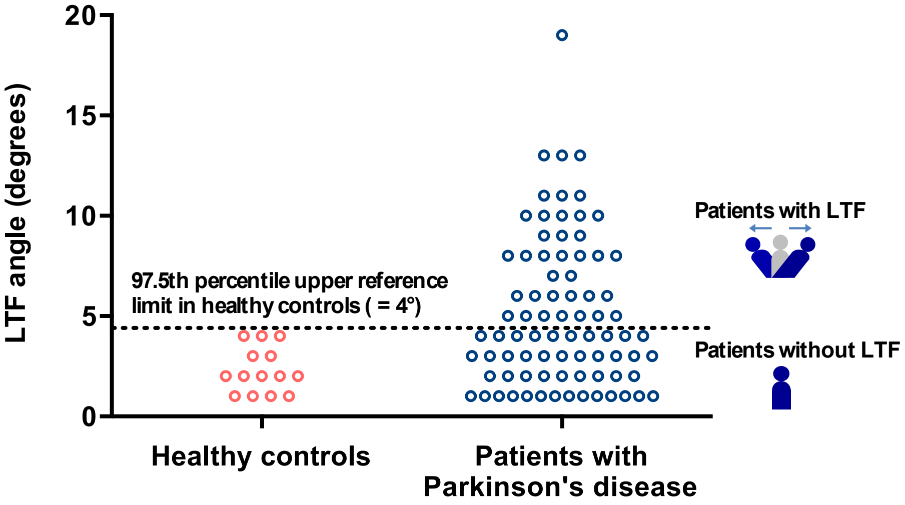

Supplementary Figure 1. Distributions of LTF angle in healthy controls and patients with Parkinson's disease. The 97.5th percentile upper reference limit of the LTF angle (degree) in the healthy controls (*n* = 14) was 4° (dotted line). Parkinson's disease patients with LTF angle > 4° were defined as patients with LTF (*n* = 37) and those with LTF angle ≤ 4° as patients without LTF (*n* = 44). LTF, lateral trunk flexion.

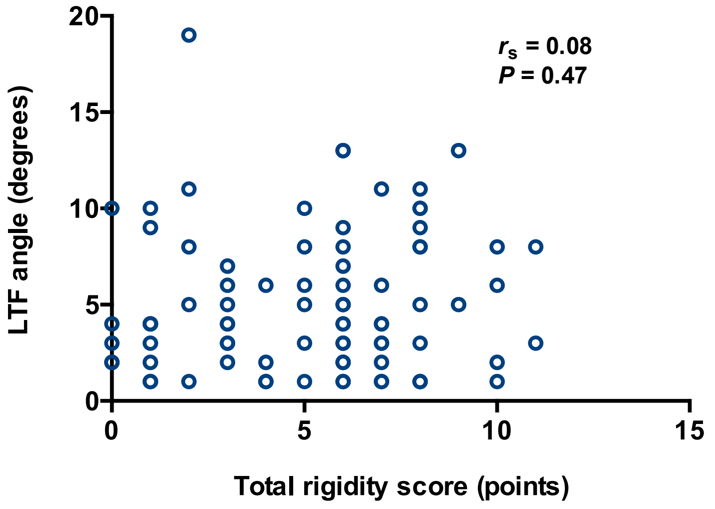

Supplementary Figure 2. Correlation between the degree of rigidity and LTF angle. There was no correlation between the total rigidity score (point) in UPDRS-III and LTF angle (degree) in patients with Parkinson's disease (n = 81). LTF, lateral trunk flexion; UPDRS-III, Unified Parkinson's Disease Rating Scale part III.

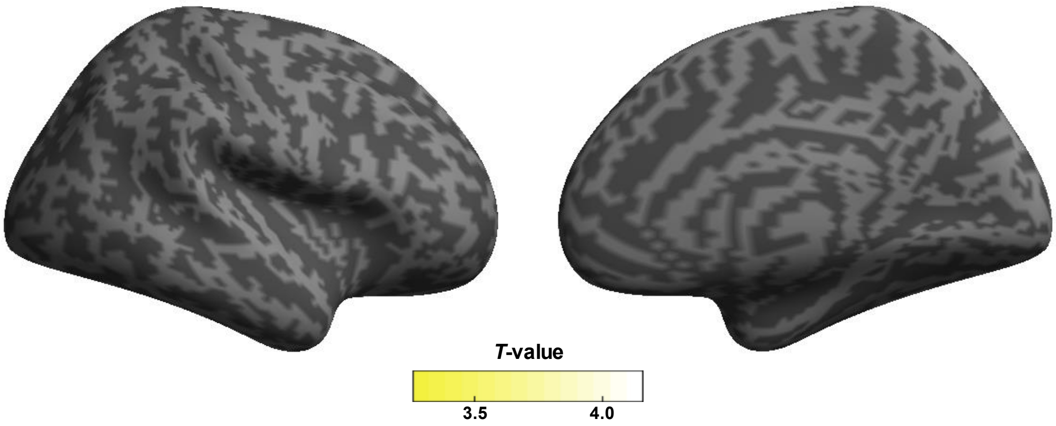

Supplementary Figure 3. Distributions of hypoperfusion in Parkinson's disease patients with LTF analyzed with family-wise error correction. No voxels survived family-wise error correction at $P < 0.05$. LTF, lateral trunk flexion.
